# The Role of the Innate Immune System in Cancer Dormancy and Relapse

**DOI:** 10.3390/cancers13225621

**Published:** 2021-11-10

**Authors:** Noah M. Chernosky, Ilaria Tamagno

**Affiliations:** 1 Department of Pathology, School of Medicine, Case Western Reserve University, Cleveland, OH 44106, USA; nmc71@case.edu; 2 Case Comprehensive Cancer Center, School of Medicine, Case Western Reserve University, Cleveland, OH 44106, USA

**Keywords:** metastasis, dormancy, immune surveillance, circulating tumor cells, macrophages, neutrophils, NK cells, MDSC

## Abstract

**Simple Summary:**

Overall survival of patients with cancer is dependent on the success of therapy. Therapy failure is correlated with enhanced metastasis and recurrence of the primary tumor. However, metastases may develop before the detection of a primary tumor and become dormant at their secondary site, presenting a major clinical challenge as these dormant cells can reactivate after the completion of seemingly successful therapy. Research has demonstrated that the innate immune system plays an integral role in molecular crosstalk with cancer cells to facilitate metastatic dissemination and control over a dormant cell state. Here, we discuss which types of innate immune cells are engaged in this crosstalk at each stage of the metastatic cascade. We also highlight how different subtypes of innate immune cells induce dormancy in cancer cells and facilitate the emergence from a dormant state. Lastly, we examine current therapeutic strategies aimed at inhibiting immune-mediated metastasis and dormancy.

**Abstract:**

Metastatic spread and recurrence are intimately linked to therapy failure, which remains an overarching clinical challenge for patients with cancer. Cancer cells often disseminate early in the disease process and can remain dormant for years or decades before re-emerging as metastatic disease, often after successful treatment. The interactions of dormant cancer cells and their metastatic niche, comprised of various stromal and immune cells, can determine the length of time that cancer cells remain dormant, as well as when they reactivate. New studies are defining how innate immune cells in the primary tumor may be corrupted to help facilitate many aspects of dissemination and re-emergence from a dormant state. Although the scientific literature has partially shed light on the drivers of immune escape in cancer, the specific mechanisms regulating metastasis and dormancy in the context of anti-tumor immunity are still mostly unknown. This review follows the journey of metastatic cells from dissemination to dormancy and the onset of metastatic outgrowth and recurrent tumor development, with emphasis on the role of the innate immune system. To this end, further research identifying how immune cells interact with cancer cells at each step of cancer progression will pave the way for new therapies that target the reactivation of dormant cancer cells into recurrent, metastatic cancers.

## 1. Introduction

Every year, over 600,000 patients succumb to cancer [1], with 90% of them having aggressive metastatic disease [2]. Often, metastatic disease returns after a successful response to treatment, indicating that some cancer cells survive and remain dormant until conditions allow for their outgrowth. Metastatic dissemination represents a major challenge in oncology; however, the mechanisms underlying tumor cell dissemination, dormancy, and relapse are still mostly unclear. Until recently, metastatic dissemination was considered a stepwise process that occurred only at the later stages of tumor progression. Over the past few years, however, the metastatic process has been revisited, with data suggesting that tumor cells can escape from a primary tumor, even before the tumor is clinically detectable: a process known as early dissemination with late metastasis. Studies monitoring the onset and development of colorectal and breast cancer showed that dissemination in less aggressive forms of cancer indeed occurred later in disease progression, thus fitting the more classic paradigm of late dissemination and increasing the overall survival of patients whose tumors were detected early. Conversely, in patients diagnosed with more aggressive subtypes of cancer, despite a substantial increase in the detection of small tumors, early diagnosis did not translate into a significant reduction in cases that eventually developed into metastatic disease [3,4,5,6].

In pre-clinical models of breast cancer, tumor cells can seed within secondary organs at early stages of the disease, undergo dormancy, and re-awaken later to drive metastatic disease [7]. In mice bearing advanced melanoma, metastatic tumor cells displayed early genetic divergence and contained unique mutations when compared to the tumor of origin, thus supporting the hypothesis of early dissemination and subsequent parallel transformation [8]. A study designed to track metastatic cells in a mouse model of pancreatic cancer showed that metastatic dissemination can occur unexpectedly early, even before the detection of a primary tumor [9]. Dormancy, therefore, plays a fundamental role in metastatic relapse, as dormant cancer cells can survive for years before they are reactivated to drive recurrent, metastatic disease. For the most part, the pattern of reactivation of dormant tumor cells appears to be unpredictable, with tumors recurring as advanced metastatic disease decades after successful treatment. Up to 20% of breast and 45% of prostate cancer patients develop metastatic disease 10 years or more after successful eradication of the primary tumor [10,11]. It is now well documented that migratory tumor cells find an environment that supports a prolonged dormant state in several tissues, including Bone Marrow (BM) [12], microvasculature of metastatic sites [13], and lymph-nodes [14], depending on the cancer subtype.

Evolving concepts of dormancy are providing clarity to two major challenges in oncology: asymptomatic minimal residual disease (MRD) [15] and cancers of unknown primary (CUP) [16,17]. MRD is asymptomatic and often undetectable; however, small clusters of residual tumor cells remain within secondary sites throughout the body and—upon specific stimuli, including increasing chronic inflammation and immune suppression—can emerge as recurrent, metastatic disease. Patients with CUP present with clearly defined metastatic lesions without having been previously diagnosed with cancer. Among the mechanisms responsible for the control of dormancy, immune surveillance represents one of the most studied, as the immune system has been shown to support tumor cells intravasation and migration towards secondary sites. Dormant cancer cells can be present either as quiescent individual cells or as small clusters, the expansion of which is prevented by immune surveillance, resulting in no net growth of the clinically undetectable micro-metastases, and the prevention of recurrent disease [18]. This review describes the main concepts concerning the role of the innate immune system in regulating cancer cell dissemination, establishment of a dormant phenotype, and reactivation of dormant cells to eventually drive advanced metastatic disease.

## 2. Key Innate Immune Cell Subtypes Involved in Metastatic Dissemination and Dormancy

This review focuses on the major subsets of innate immune cells involved in the metastatic cascade and that mediate dormancy of disseminated tumor cells (DTCs), including Tumor-Associated Macrophages (TAMs), Tumor-Associated Neutrophils (TANs), Natural Killer (NK) cells, and Myeloid-Derived Suppressor Cells (MDSCs). Cells within these lineages become polarized to more specific subtypes by the growth factors, cytokines, and chemokines within the tumor microenvironment (TME). For example, TAMs can undergo classical polarization to an anti-tumorigenic M1 phenotype via Interferon-γ (IFN-γ), Tumor Necrosis Factor α (TNF-α), or Lipopolysaccharide (LPS) stimulation. TAMs can alternatively be polarized to a pro-tumorigenic M2 phenotype via Interleukin-4 (IL-4), IL-10, or IL-13 stimulation [19]. Similarly, TANs can also be polarized to an anti-tumorigenic N1 phenotype via type I interferon stimulation [20] or to a pro-tumorigenic N2 phenotype via Tumor Growth Factor β1 (TGF-β1) stimulation [21]. In the case of MDSCs, both subsets—polymorphonuclear MDSCs (PMN-MDSCs) and monocytic MDSCs (M-MDSCs)—are pro-tumorigenic. The distinction between PMN-MDSCs and M-MDSCs is that PMN-MDSCs mature from granulocytic precursors via Granulocyte Macrophage Colony-Stimulating Factor (GM-CSF), IL-6, or IL-1β stimulation while M-MDSCs mature from monocytic precursors via Macrophage Colony-Stimulating Factor (M-CSF) stimulation [22].

Both M1 TAMs and N1 TANs demonstrate anti-tumorigenic phenotypes through Antibody-Dependent Cellular Cytotoxicity (ADCC) of burgeoning tumor cells. Additionally, M1 TAMs can phagocytize tumor cells through direct physical interactions, while N1 TANs can indirectly regulate phagocytosis of tumor cells through the secretion of the proinflammatory cytokines TNF-α and IL-12 [21,23]. However, the pro-tumorigenic subtypes of these cells—as well as both PMN-MDSCs and M-MDSCs—aid the tumor in several steps of the metastatic cascade and dormancy. Below we detail how these subtypes of innate immune cells contribute to each step of the metastatic cascade and induce dormancy at distant, metastatic sites.

## 3. Immune Regulation of the Metastatic Process

### 3.1. Intravasation

There are three major conditions that must occur to allow for tumor cell intravasation: angiogenesis, Epithelial-Mesenchymal Transition (EMT) of the tumor cells, and suppression of anti-tumorigenic immune cells. Tumor-secreted GM-CSF, CXCL12, and CCL2 attract monocytes to the TME and drive their differentiation into M2 TAMs, which then promote angiogenesis through secretion of Matrix Metalloproteinase 9 (MMP9) and Vascular Endothelial Growth Factor A (VEGF-A) [23,24,25,26,27,28,29]. Major Histocompatibility Complex (MHC) Class I expressed on the surface of tumor cells engages with Leukocyte Immunoglobulin Like Receptor B1 (LILRB1) on the surface of M2 TAMs to inhibit macrophage phagocytosis of the tumor cells [30].

Similarly, CXCL5, CXCL6, and CXCL8 recruit neutrophils to the TME, and tumor-secreted TGF-β1 polarizes them to an N2 phenotype [21,31,32]. N2 TANs facilitate angiogenesis via upregulation of MMP9 and secretion of VEGF-A and Oncostatin M (OSM) [21,33]. Furthermore, M2 TAMs and N2 TANs are responsible for facilitating EMT and remodeling the Extracellular Matrix (ECM) of the tumor cells, both of which convert tumor cells to a more migratory phenotype. M2 TAMS drive tumor cell EMT via GM-CSF and CCL18 [23,29] while N2 TANs remodel the ECM of tumor cells via Neutrophil Elastase (NE) and MMP9 [33]. Additionally, tumor-secreted factors, including IL-6 and OSM, activate Signal Transducer and Activator of Transcription 3 (STAT3) within M-MDSCs, which, in turn, drive a mesenchymal and invasive phenotype in the tumor cells [34,35]. It is currently unclear if PMN-MDSCs mitigate the EMT of tumor cells. Additionally, it remains to be seen if either MDSC subset facilitates angiogenesis at the primary tumor.

Equally important in the facilitation of tumor cell intravasation is the suppression of pro-inflammatory, anti-tumorigenic immune cell functions. NK cells are important anti-tumorigenic cells at the primary tumor and secondary sites that prevent tumor growth by their phagocytizing of Cancer Stem Cells (CSCs) [3,36]. M2 TAM secretion of TGF-β1 exhausts anti-tumorigenic NK cells, effectively suppressing their cytotoxicity against the tumor cells [37]. M2 TAMs also impact adaptive immune anti-tumorigenic functions by recruiting regulatory T cells (T_regs_) to the TME, which in turn inhibit effector T-cell functions [23]. N2 TANs are likewise capable of inhibiting cytotoxic T-cell functions through upregulation of inducible Nitric Oxide Synthase (iNOS) and increased secretion of Arginase 1 (Arg1) [21,33,38]. MDSCs in general also suppress innate and adaptive anti-tumorigenic mechanisms. MDSCs inhibit NK-cell-mediated ADCC by repressing the Fc receptor on the surface of NK cells [39] and secrete TGF-β1 to inhibit adaptive immune responses via effector T-cell function, causing expression of Programmed cell Death-1 (PD-1) on the surface of Tumor-Infiltrating Lymphocytes (TILs) [40]. Immune suppression at the primary tumor, along with increased angiogenesis and the development of a migratory tumor cell phenotype, affords enhanced accessibility for tumor cells to invade into circulation.

### 3.2. Survival in Circulation

Once tumor cells intravasate into the bloodstream, they require assistance to survive and successfully travel to a secondary site for eventual colonization. The physical turbulence of pulsing blood and the presence of anti-tumorigenic immune cells are two major obstacles to the metastatic cascade. N2 TANs support Circulating Tumor Cells (CTCs) by forming neutrophil-cancer cell clusters that facilitate cancer cell survival while in the bloodstream and escort them toward secondary tissues for seeding [41]. Neutrophils create clusters with CTCs by forming Neutrophil Extracellular Traps (NETs), large extracellular structures made of a chromatin scaffold providing anchorage to neutrophil-derived enzymes, such as elastase and myeloperoxidase [42]. Cathepsin C, for example, has been demonstrated to recruit neutrophils to the primary tumor and induce NETs that enhance tumor cell dissemination [43]. Nanoparticle-mediated depletion of NETs reduces pulmonary metastases in a breast cancer model, demonstrating that targeting neutrophil-assisted tumor cell dissemination is a potential therapeutic strategy [44]. While TANs are historically known for forming clusters around CTCs, emerging research demonstrates that MDSCs can serve a similar function. In particular, PMN-MDSCs have been shown to form clusters with CTCs to support their survival in the bloodstream via Reactive Oxygen Species (ROS)-dependent activation of Notch1 ligands [45]. It is unclear if M-MDSCs also form clusters with CTCs because current research is insufficient.

The suppression of NK-cell functions is an important aspect for the survival of CTCs in circulation. While facilitating transit of CTCs within the bloodstream, N2 TANs also inhibit NK-cell-mediated phagocytosis of CTCs, effectively mediating survival of CTCs until they extravasate to a secondary tissue [46]. CTCs themselves also directly inhibit NK-cell function through upregulation of NK cell inhibitory receptor ligands, such as Programmed Death Ligand 1 (PD-L1) and Human Leukocyte Antigen G (HLA-G). In addition, CTCs indirectly repress NK-cell function through secretion of IL-10, TGF-β1, and other immunosuppressive cytokines that inhibit NK-cell-mediated killing via MDSC and T_reg_ activity [3]. In particular, PMN-MDSCs demonstrate suppressive effects on both NK cells and anti-tumorigenic adaptive immune cells [45].

### 3.3. Extravasation at Distant Sites

Extravasation into a secondary tissue is the last step of the metastatic cascade. Extravasation is mediated both by the innate immune cells that chaperone CTCs in the bloodstream and by innate immune cells that form a pre-metastatic niche receptive to the migrating tumor cell(s). Similar to their role in the intravasation step, M2 TAMs enhance CTC movement into a secondary tissue through activation of C-C chemokine receptor 1 (CCR1). CCR1 mediates the formation of complexes between M2 TAMs and CTCs, thus prolonging their interactions and thereby increasing the chances of CTCs to successfully seed secondary organs. Furthermore, N2 TANs secrete MMP8, MMP9, and IL-1β to prime endothelial cells to accelerate the process of extravasation [46]. In turn, activated endothelial cells expressing the Notch1 receptor increase the expression of Vascular Cell Adhesion Molecule 1 (VCAM1), which allows for direct interactions between endothelial cells and CTCs that further facilitate extravasation [47].

**Figure 1:**
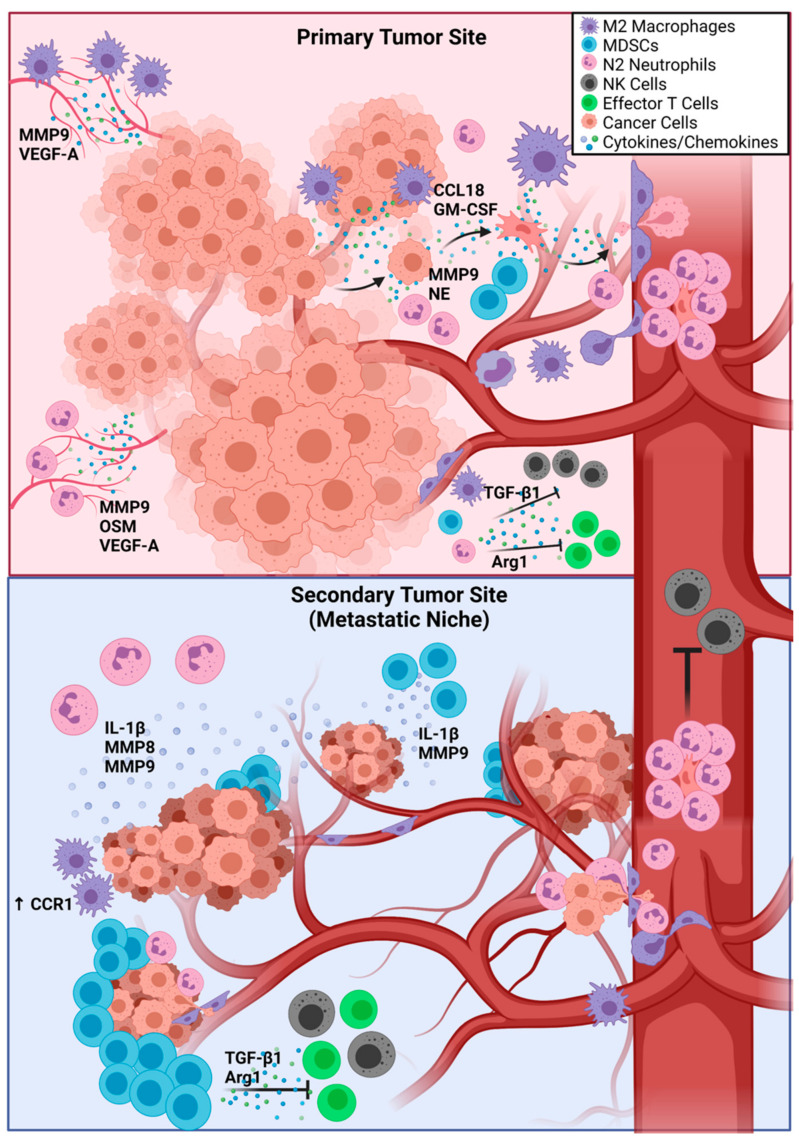
The Influence of Innate Immune Cells on Metastasis. M2 TAMs and N2 TANs drive angiogenesis at the primary tumor via secretion of MMP9, VEGF-A, and OSM. M2 TAMs secrete CCL18 and GM-CSF to drive EMT in tumor cells, while N2 TANs remodel the ECM of tumor cells through secretion of MMP9 and NE. M2 TAMs, N2 TANs, and MDSCs secrete TGF-β1 and Arg1 to inhibit the functions of NK cells and effector T cells. In the bloodstream, N2 TANs form NETs around tumor cells to protect them and guide them to a secondary site. At the secondary site, activation of CCR1 in M2 TAMs and secretion of IL-1β, MMP8, and MMP9 by N2 TANs primes endothelial cells for extravasation. MDSCs facilitate extravasation through secretion of IL-1β and MMP9 and create an immunosuppressive environment through secretion of TGF-β1 and Arg1. Abbreviations: TAMs—tumor-associated macrophages; TANs—tumor-associated neutrophils; MMP8 and MMP9—matrix metalloproteinase 8 and 9; NE—neutrophil elastase; VEGF-A—vascular endothelial growth factor A; OSM—Oncostatin M; GM-CSF—granulocyte-macrophage colony-stimulating factor; EMT—epithelial-to-mesenchymal transition; MDSCs—myeloid-derived suppressor cells; TGF-β1—transforming growth factor β1; Arg1—Arginase 1; NETs—neutrophil extracellular traps; IL-1β—Interleukin 1β.

Both PMN-MDSCs and M-MDSCs support the establishment of a pre-metastatic niche by repressing both innate and adaptive anti-tumor responses driven by NK cells and effector T cells, respectively [39,40]. PMN-MDSCs and M-MDSCs have been shown to form pre-metastatic niches in the lungs of melanoma-bearing mice [48]. PMN-MDSCs significantly increase the permeability of vascular endothelial cells in the lungs through secretion of MMP9, therefore facilitating CTC extravasation. Importantly, nanoparticle-mediated disruption of PMN-MDSC adhesion to the vascular endothelium can effectively prevent metastases, demonstrating a therapeutic avenue for targeting pre-metastatic niches [49]. Similarly, M-MDSCs are recruited to the lungs by tumor-secreted CCL12 and drive tumor cell adhesion to the vascular endothelium through secretion of IL-1β [50]. Once the CTCs have seeded a pre-metastatic niche, they generally become dormant until they can adapt to the new environment and overcome immune surveillance. Figure 1 displays the metastatic cascade from primary tumor intravasation to extravasation at a secondary site, with an emphasis on the role of innate immune cells at each step. Emerging research suggests that chemotherapy-induced inflammation actually increases metastatic potential in secondary organs [51], opening up new avenues of research aimed at understanding mechanisms of metastasis following therapy failure.

## 4. Promotion of Dormancy by the Immune System

The cytotoxic and cytostatic functions of specific immune subtypes located within secondary sites induces immunogenic dormancy, which prevents the development of micro-metastases as well as the progression of metastatic disease. The immunologic equilibrium theory states that immune surveillance suppresses the proliferation of tumor cells, which will reach their dormant state through the acquisition of a reversible growth arrest and selective reduction of their immunogenic potential. Immunologic equilibrium will stand until new mutations in the tumor cells and/or further immune suppression will set the stage for immune escape and development of metastatic disease [52]. Preclinical models of breast cancer and melanoma have provided crucial clues about the mechanisms of early metastatic dissemination and dormancy [8]. Depletion of NK cells in models of lung and breast cancer appears to create favorable conditions for metastatic disease [53]. When the NK-cell population is depleted, quiescent cells reactivate their proliferation cycle and drive macro-metastatic lesions in secondary organs. The most common immune evasion mechanisms are the ER stress/misfolded protein pathway, which causes downregulation of Major Histocompatibility Complex I (MHC I), thus repressing antigen presentation to the immune cells [54]; upregulation of the immune checkpoint inhibitor PD-L1 that drives apoptosis in adaptive immune cells; and EMT [55], usually associated with chronic inflammation.

Similarly, in a 2016 study using advanced melanoma models focusing on adaptive immune response, researchers observed late metastatic disease (over 200 days post-engraftment), predominantly within the lungs. Secondary lesions, however, presented a significantly distinct Single Nucleotide Polymorphism (SNP) profile, with only a few mutations shared between primary and metastatic tumors. These data suggest that the lung metastases derived from developing cancer cells that had migrated early in the transformation process seeded into the lungs and, later on, developed independent metastatic lesions. DTCs within the lungs demonstrated a low proliferation index in the absence of apoptosis, thus suggesting their engagement in a dormant state and a latency in tumor growth due to lack of proliferation. CD8^+^ T cells patrolling the lungs were responsible for inducing dormancy in migratory tumor cells, thereby delaying the onset of metastatic disease. Depletion of CD8^+^ T cells led to more rapid development of metastatic lesions, with more and larger metastatic nodules detected within the lungs [8].

Repression of MHC I is a common mechanism of escape from an immune response that CTCs adopt, mainly to avoid triggering adaptive immunity [56]. As a matter of fact, the frequency of Tumor-Infiltrating Lymphocytes (TILs) appears to be reduced in tumors with low expression of MHC I, therefore impacting response to T-cell-based immunotherapy (CAR-T—Chimeric Antigen Receptor-T cells, Immune Checkpoint Inhibitors—ICIs). Although mostly responsible for activating adaptive immunity, MHC I is also upregulated during the first phase of innate responses, and its downregulation can prevent CTCs from being targeted by innate immune cells [57].

Not surprisingly, the ability of cancer cells to evade the immune response correlates with their metastatic potential and aggressiveness. In a syngeneic model of non-metastatic triple-negative breast cancer, disseminated EMT6 cells were completely eradicated by circulating CD8^+^ T cells, thus suggesting higher susceptibility to immune recognition and response. On the other hand, D2A1 cells, highly metastatic to the lungs, are induced by patrolling immune cells to enter a dormant state and survive in a status of immune equilibrium, which prevents them from forming large metastatic nodules while at the same time protecting them from immune surveillance until they are able to revert their dormant state and actively form metastatic nodules [58,59]. Although these influential studies have provided a foundation for cancer dormancy research, there is a limited amount of research dedicated to cancer cell dormancy. This is likely because studying dormancy is difficult due to the quiescent state of dormant cells as well as a lack of knowledge on the mechanisms that induce dormancy, making it challenging to generate animal models that recapitulate this process and demonstrate consistent results.

## 5. Immune-Mediated Escape from Dormancy

Having established that the micro-metastatic niche—comprised of tumor cells, immune cells, and other tissue stromal cells—creates a new and unique microenvironment, we will next discuss how the evolution of the dormancy-associated microenvironment can lead to the reactivation of cancer cell expansion and macro-metastatic outgrowth. Several factors released within the TME exert opposing actions: either mediating or reverting dormancy. Depending on the subtype of solid tumor, dormancy can be prevented or reverted upon secretion of TGF-β1 and Periostin (POSTN) [13] by sprouting endothelial cells. Conversely, IFN-γ secreted by CD4^+^ and CD8^+^ T cells can induce dormancy, together with TGF-β2 and Bone Morphogenetic Proteins (BMPs). Neutrophil-mediated proteolysis of Thrombospondin 1 (TSP-1), which in its full-length form prevents reactivation of dormant cells, can also contribute to escape from dormancy in response to chronic inflammation [60].

While chronic inflammation is well known to promote the onset and development of cancer, it is now also being implicated as one of the main drivers capable of re-awakening dormant cancer cells. Patients suffering from chronic inflammation are more likely to develop advanced metastatic disease as a consequence of the secretion of pro-inflammatory cytokines such as TNF-α, IL-10, IL-1β, IL-8, TGF-β1, IL-18, and members of the IL-6 family, including OSM [35,61,62]. IL-6 and IL-8 levels correlate with increases in MDSCs, M2 TAMs, and N2 TANs, which establish immune tolerance by secreting TGF-β1 and IL-10 [18]. IL-18 secreted by cells undergoing EMT promotes the expression of PD-1 on NK cells, thus mediating their inactivation upon binding with PD-L1 expressed on the surface of tumor cells [63]. Studies in preclinical models of pancreatic cancer demonstrated that CTCs underwent EMT before further invading secondary organs [9,64]. Interestingly, upon treatment with immunosuppressive drugs, such as dexamethasone, inflammation and invasiveness were contained [9]. EMT has recently been associated not just with higher invasiveness in tumor cells but with the preparation of a pro-tumorigenic immune microenvironment in the secondary organs [65,66]. The ability of breast cancer cells to become more invasive after EMT relies, in part, on their ability to suppress immune cell function by secreting the aforementioned cytokines. TGF-β1, BMPs, IL-6, and other IL-6 family members activate immune-suppressive signaling in both tumor cells and immune cells. For example, in response to EMT-mediating inflammatory cytokines, the transcription factor Runt-related Transcription Factor 3 (RUNX3) binds to the Forkhead Box P3 (FOXP3) promoter and increases the T_reg_ population within the tumor. T_regs_ act by regulating both innate and adaptive anti-tumorigenic immune responses and mediating the establishment of immune evasion [67].

Neutrophils are among the cells of the innate immune system to mediate chronic inflammation in patients and have a specific role in facilitating the re-awakening of dormant cells. Sustained experimental lung inflammation in mice, through either intra-nasal instillation of LPS or prolonged exposure to smoke, causes the reactivation of dormant breast cancer cells seeded in the lungs [68]. ECM remodeling triggered by neutrophils can activate Integrin α5β1-mediated signaling within dormant cells, thus promoting their proliferation. Integrin α5β1 has been previously associated with awakening dormant cells through its activation of Extra-Cellular Signal-Regulated Kinase (ERK) [69,70]. ERK signaling is inhibited in dormant cells and is counterbalanced by strong activation of the p38 Mitogen-Activated Protein Kinase (MAPK); the ERK-p38 balance is an important determinant of dormancy. Upon Integrin α5β1 activation, p38 is inhibited, and ERK is activated, resulting in the resumption of proliferation [14]. However, there is insufficient research dedicated to identifying what activates p38 to maintain a state of quiescence in dormant DTCs.

## 6. The Molecular Crosstalk between Cancer Cells and the Tumor Microenvironment

Tumor cells have preferred routes of dissemination and preferred sites of metastasis, depending on tumor type and the treatments received by the patients. One of the first models of tumor cell dissemination was proposed by Stephen Paget in 1899. According to his “Seed and Soil Hypothesis”, cancer cells are envisioned as seeds requiring the right soil to grow [71,72]. Therefore, interactions with neighboring cells and specific signaling events, mediated by cytokines and growth factors, are essential for metastatic cells to colonize secondary organs. For example, CXCR4 facilitates the binding of breast cancer cells to endothelial cells at metastatic sites through a mechanism similar to that observed with leukocyte intra- and extravasation. CXCR4 expression promotes breast cancer cell migration towards sites of potential metastasis that actively secrete CXCL12, the ligand of CXCR4, whose production is increased during tumor-promoting processes, such as angiogenesis and inflammation [7,73]. Furthermore, CXCL12 is known to maintain leukemia stem cells in a quiescent state within the bone marrow, protecting them from therapy [74].

Metastatic cells seed different tissues; however, only specific regions within the secondary sites function as specialized niches for dormant cells, including BM with low osteoclast activity [75,76], stable microvasculature [13], and lymph nodes [77,78]. However, there is insufficient data regarding specific homing mechanisms that regulate the seeding of dormant cells within a specific niche. DTCs are maintained in their immune-mediated dormant state within these niches by factors secreted by the surrounding microenvironment, including BMP4 and TGF-β2 [79]. Conditions affecting the homeostasis of these niches—including chronic inflammation, hypoxia, and lesions—cause tissue remodeling that can trigger the reactivation of the dormant cells through the secretion of factors such as TGF-β1, BMPs, and cytokines secreted within dormant cell niches during the restoration of homeostasis [13]. While osteoblasts support DTC dormancy via secretion of TGF-β2, it has been suggested that BMP4- (more abundantly found in the bones of younger subjects) and Growth Differentiation Factor 10 (GDF10)-positive osteoclasts can trigger their re-awakening and therefore mediate the formation of osteolytic metastatic nodules within the bone [80]. DTC-mediated bone resorption and the associated inflammatory microenvironment require the binding of the Receptor Activator of Nuclear Factor kappa-B Ligand (RANKL) to its receptor RANK expressed by osteoblasts, that drives secretion of TGF-β1, thus mediating reactivation of dormant cells and the formation of micro-metastases [81].

**Figure 2:**
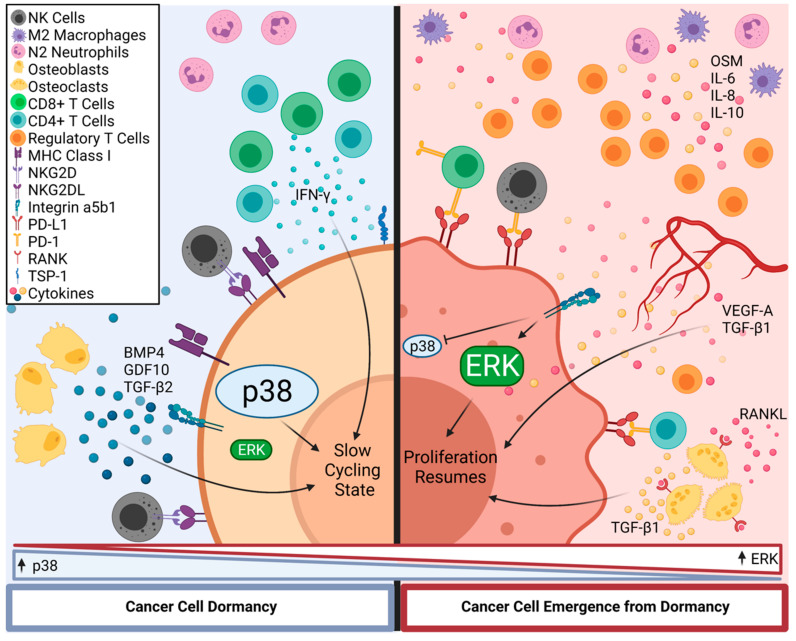
Control of Disseminated Tumor Cell Dormancy by Innate Immune Cells. After successful extravasation at a secondary site, DTCs become dormant, characterized by increased p38 activity and expression of MHC Class I molecules and TSP-1 on the cell surface. Effector T-cell-secreted IFN-γ and osteoblast-secreted BMP4, GDF10, and TGF-β2 maintain dormancy within DTCs. Cleavage of TSP-1, expression of PD-L1, and Integrin α5β1-driven activation of ERK are characteristic of DTCs emerging from dormancy. Secretion of OSM, IL-6, IL-8, and IL-10 from M2 TAMs and N2 TANs drives T_reg_ expansion and an immunosuppressive environment to allow for survival of DTCs emerging from dormancy. DTC proliferation is also stimulated as a result of VEGF-A and TGF-β1 secretion. The expression of NKGD2L on tumor cells protects them from NK cells by driving their exhaustion. Abbreviations: DTCs—disseminating tumor cells; MHC I—major histocompatibility complex I; TSP-1—thrombospondin 1; IFN-γ—Interferon g; BMP4—bone morphogenetic protein 4; GDF10—growth differentiation factor 10; TGF-β2—transforming growth factor β2; PD-L1—programmed death-ligand 1; OSM—Oncostatin M; IL-6, -8, and -10—Interleukin 6, 8, and 10; TAMs—tumor-associated macrophages; TANs—tumor-associated neutrophils; VEGF-A—vascular endothelial growth factor A; TGF-β1—transforming growth factor β1.

Micro-metastatic clusters of breast cancer cells tend to seed within the Perivascular Niche (PVN), either within the bone or in different organs, such as lungs [13]. The reactivation of dormant cells is often associated with angiogenesis-triggering stimuli like hypoxia and inflammation. While established endothelial cells do not perturbate the dormant state of cancer cells, newly sprouting micro-vasculature secrete factors such as VEGF-A and TGF-β1 [82], which are associated with tumor cell proliferation and immune suppression. Lymph nodes also provide routes for tumor cell dissemination, sheltering dormant cells, and providing access to distant organs [78]. Due to their physiological role, lymph nodes are involved in most processes related to acute and chronic inflammation as well as inflammatory responses triggered by cancer. It has been suggested that several inflammatory stimuli can reactivate dormant cells within distant nodes. One of the main mediators of dormant cell reactivation within lymph nodes is the transcription factor Zinc Finger E-box-Binding Homeobox 1 (Zeb1), a promoter of EMT in response to inflammation [83]. Figure 2 depicts the differences in receptors expressed on dormant and proliferating cells, as well as the conditions in the TME that control the dormancy status of a DTC. There is much research that remains to be done to identify specific markers of dormancy that can be exploited for therapy.

## 7. Therapeutic Avenues

Therapeutic targeting of both disseminating and dormant tumor cells could be an efficacious strategy to overcome resistance to standard therapy and development of advanced metastatic disease. Several components of the Tumor Immune Microenvironment (TIME), including dormant cell niches within metastatic sites, have thus far been considered as potential candidates for either diagnosis or treatment of metastatic malignancies.

### 7.1. Liquid Biopsy and Advanced Flow Cytometry

Immuno-oncology is gaining momentum and will require new paradigms not just for the treatment but also for the diagnosis and follow-up of cancer patients, taking into consideration specific cell types within the TME, in particular immune cells, either within the primary and secondary tumors or in circulation. To this end, Liquid Biopsies (LBs) and advanced flow cytometry techniques will become increasingly important for the monitoring of tumor and immune cells in circulation as well as of their activation state, which could predict response to therapy and outcome in more aggressive types of cancer.

LB represents the future of more traditional tools for the detection of specific cell markers in patient-derived samples. For example, detection of PD-L1 expression in Non-Small Cell Lung Cancer (NSCLC) patients via immunohistochemistry (IHC) has been routinely used to identify potential candidates for PD-L1 inhibitor-based treatment [84]. LB can provide information about the response, relapse, and adverse effects for patients with cancer undergoing immune-based therapies. CTCs, exosomes, cytokines, and proteins, as well as cell-free DNA (cf-DNA), can be collected and further analyzed. Most importantly, the frequency and activation status of immune subtypes involved in either supporting or blocking tumor growth and dissemination can be evaluated, including innate immune cells and T cells, mostly post-CAR-T- and immune checkpoint inhibitors-based treatments [85,86]. High-parameter flow cytometry analysis of LB allows the detection of numerous markers of cell type and activation status simultaneously. Whole-blood multi-parametric flow cytometry analysis of patients with breast cancer provided valuable insights on the frequency and phenotype of specific innate and adaptive immune subtypes. In particular, a positive correlation was identified between a drop in the secretion of IFN-γ by NK cells and an increased risk of metastatic disease. This is due to an alteration in crosstalk between NK cells and dendritic cells (DC) and monocytes, which prevents the activation of an efficacious anti-tumorigenic immune response. Altered secretion of TNF-α in CD14^low^-CD16^+^ monocytes was also correlated with a compromised ability to convert into Monocyte-derived Dendritic Cells (MoDCs) or M1 TAMs [87]. More recently, high-parameter flow cytometry has been employed for monitoring the CAR-T applications in B-cell leukemia and lymphomas [88]. Besides assessing the frequency of CAR-T cells versus the frequency of CD19^+^ B cells in blood samples from patients, Blache and colleagues focused on the frequency and activation status of NK cells, including the expression of the activating receptor Natural Killer Group 2, Member D (NKG2D) and the cytotoxicity receptor Natural Killer protein 46 (NKp46). This type of flow cytometry analysis could be used for patients undergoing Chimeric Antigen Receptor-NK (CAR-NK)-based treatments.

### 7.2. NK Cells

NK cells were first identified for their ability to recognize and attack tumor cells without antigen priming [89], placing them among the first and most efficient responders to a developing tumor or disseminated tumor cells. NK cells are excellent candidates for activating cell therapies aimed at depleting primary or metastatic dormant tumor cells in various types of solid tumors. Among the most promising applications of NK cells in the treatment of metastatic cancer are NKG2D engagement [90] and CAR-NK cells. The ligands of NKG2D, NKG2D-L, are expressed on the surface of stressed or infected cells to attract NK cells and cause their activation. Dormant breast and lung cancer cells express elevated NKG2D-L causing exhaustion in NK cells [91]. Targeting NKG2D-L-expressing tumor cells could represent an efficacious avenue to reactivate the NK response against dormant cells. Alternatively, CAR-NK cells targeting markers of dormancy represent a potential therapeutic tool by either complementing the adaptive anti-tumor response of cytotoxic CD8^+^ cells or by offering an alternative for cancers unresponsive to T-cell-based therapies. Like CAR-T cells, CAR-NK cells are generated to express receptor proteins allowing the recognition of tumor-specific antigens and to mediate secondary signaling. CAR-NK cells offer several advantages in comparison to CAR-T cells, including a lower risk of Graft-versus-Host Disease (GvHD), due to a minimal secretion of cytokines, and an easier generation from different sources, including patient-derived Peripheral Blood Mononuclear Cells (PBMCs), induced Pluripotent Stem Cells (iPSC) and immortal lines, such as NK-92 [92]. The main obstacle to successful CAR-NK- as well as CAR-T-based therapies remains the presence of heterogeneous populations within solid tumors. In particular, CTCs and dormant cells tend to modulate the expression of surface markers potentially able to trigger immune responses, therefore eluding CAR-engineered cells [93]. A recent study showed that CAR-NK cells targeting PD-L1 expressed by Head and Neck Squamous Carcinoma (HNSCC) cells can successfully overcome the establishment of immune tolerance and eliminate tumor cells [94]. The majority of clinical trials approved for CAR-NK cells are focused on hematologic malignancies rather than metastatic solid tumors, with only one trial currently evaluating the response of castration-resistant prostate cancer patients to CAR-NK cells targeting Prostate-Specific Membrane Antigen (PSMA) [95]. Although CAR-NK cells show mild side effects and initial efficacy in lymphoid cancer patients [96], further studies are needed to evaluate short- and long-term effects of CAR-NK-based treatments in patients affected by advanced metastatic disease.

### 7.3. Targeting Integrin Signaling to Prevent Re-Awakening of Dormant Tumor Cells

As previously described, neutrophils trigger reactivation of dormant cells by driving ECM remodeling during chronic inflammation. In particular, neutrophils release proteolytic enzymes from their secretory granules, including MMP9 and NE. Catabolism of ECM-associated components, in particular laminin and fibronectin, causes the activation of ECM receptors on the surface of dormant cancer cells through Integrin β1, thus driving activation of ERK [10,67,68]. Integrin signaling is becoming increasingly important as a potential target to prevent the reactivation of dormant cells. We have already discussed the role of Integrin α5β1 in regulating the ERK/p38 balance in response to neutrophil-driven conformational changes in the ECM [14]. Upon binding with fibronectin, Integrin α5β1 activates ERK and inhibits p38 to re-awaken dormant cells. Conversely, in normal tissue ECM, fibronectin does not undergo proteolysis: therefore, integrin α5β1 receptors are inactive, and p38 signaling is predominant. At the same time, Periostin (POSTN), which is secreted by endothelial cells during angiogenesis, functions as a ligand for both Integrin α5β3 and Integrin α5β1, driving the escape from dormancy as well as activating WNT signaling [18].

Inhibiting integrin signaling is currently under investigation as a therapeutic avenue for metastatic cancer. The efficacy of anti-Integrin α5 monoclonal antibodies has been assessed in several clinical trials, including Intetumumab (CNTO95) [97] and Abituzumab (EMD 525797/DI17E6) [98]. While it increased overall survival in metastatic melanoma patients, Intetumumab did not significantly impact the survival of subjects with hormone-refractory prostate cancer. Increasing doses of Abituzimab did not correlate to increased progression-free survival in participants with colorectal cancer metastatic to the liver, alone or in combination with Cetuximab and Irinotecan (in metastatic colorectal and ovarian cancer). Volociximab (M2000) is a monoclonal antibody specifically binding to Integrin α5β1, which has shown inhibition of angiogenesis and tumor growth in the rabbit VX2 carcinoma model [99,100]. Volociximab has been investigated in phase I clinical trials for various classes of tumors, including metastatic melanoma and metastatic pancreatic cancer, alone or in combination with bevacizumab and chemotherapy. Results from those trials are currently pending [101]. Endogenous antagonists of Integrin α5β1, such as endostatin, represent an alternative strategy for controlling the reactivation of dormant tumor cells and are currently under study for the treatment of NSCLC [102]. A similar approach, whether with monoclonal antibodies or endostatins, has been proposed for imaging purposes to diagnose and localize micro-metastases [103].

### 7.4. Reactivation of Type I and II Interferon Pathways

Type I and II interferons impinge upon tumor physiology by both affecting tumor cell proliferation and activating innate and adaptive anti-tumor immune responses through STAT1 and STAT2 activation [104,105]. This is driving drug discovery for the development of interferon-based therapies aimed at re-activating downstream effectors of interferons either endogenously or exogenously [106]. Our group has recently described the opposing effects of IFN-β versus OSM and TGF-β1 in preventing transformed mammary epithelial cells from undergoing EMT [107]. Similarly, another group identified the IRF7/IFN-β axis as a driver of dormancy in breast cancer cells following chemotherapy, with a spontaneous escape from dormancy driven by IFN-γ depletion. High levels of IFN-β are then indicative of a good prognosis in response to chemotherapy as the risk of metastatic recurrence is mitigated by the promotion of dormancy in disseminated cells [108]. Moreover, IFN-β is widely recognized for its ability to boost tumor antigen presentation [109] and to prompt the production of chemokines that attract anti-tumor immune cells [110]. In fact, IFN-β can be induced by chemotherapy and is indispensable for cytotoxic treatments to induce Immunogenic Cell Death (ICD), which accounts for substantial, if not a majority, of the therapeutic response. Stimulator of Interferon Genes (STING), an activator of type I and II interferons, drives innate immune responses against pre-existing tumors in preclinical models of triple-negative breast cancer [111]. STING activation appears to mediate the re-programing of TAMs into anti-tumorigenic M1 TAMs while reducing the M2 TAM population [112,113]. Furthermore, STING activation appears to be required for the maturation and activation of NK cells [114]. Downregulating cytokines with opposing effects to type I and II interferons could help restore innate and adaptive anti-tumorigenic immune responses as well as prevent the re-awakening of dormant cells seeded in secondary organs.

TEW-7197 (Vactosertib), an orally bioavailable inhibitor of the TGF-β1 pathway that blocks the activation of the TGF-β1 Receptor (TGFBR1), has shown promising results. When administered in combination with adoptively transferred NK cells transplanted in mouse models of melanoma, TEW-7197 caused apoptosis of tumor cells in vitro as well as tumor regression in vivo [115]. Recently, a phase I clinical trial testing TEW-7197 has started at the Jo Oh Park, Samsung Medical Center in South Korea to determine its efficacy in patients with metastatic pancreatic cancer who have failed first-line gemcitabine and paclitaxel [116]. Despite their efficacy in preventing tumor growth and eliciting pro-inflammatory immune responses in vivo, important side effects are usually associated with the systemic administration of type I interferons in oncologic patients. Symptoms such as fatigue, anorexia, flu-like and neurological manifestations can impact the quality of life of patients so significantly that often times they decide to discontinue the treatment [106,117]. More research is needed to identify type I interferon-based therapies with less impact on the patients’ well-being.

Similar to type I interferons, reactivation of IFN-γ pathways may also present a viable therapeutic strategy. Clinical studies using NSCLC patient data have demonstrated that elevated IFN-γ activity correlates to enhanced survival and better responses to therapies targeting the PD-1/PD-L1 axis [118,119]. IFN-γ demonstrates anti-tumorigenic properties through the induction of apoptosis in tumor cells as well as via induction of dormancy. However, chronic IFN-γ actually enables tumorigenesis and growth of the primary tumor, demonstrating that there must be a balance to IFN-γ activity if used as a therapeutic strategy [120]. Furthermore, the use of IFN-γ has seen mixed results in a clinical setting, with observable adverse side effects, including fever and rash, among others [121]. Similar to type I interferons, it appears that IFN-γ can be harnessed in some capacity as an anti-tumorigenic therapeutic approach, but appropriate measures must be considered to ensure its efficacy.

Importantly, reactivation of IFN-β and IFN-γ pathways can trigger an increase in MHC I expression on tumor cells, therefore facilitating their recognition by both the innate (NK cells) and adaptive (CD8^+^ cytolytic T cells) immune system. If restoring IFN signaling may cause important side effects in patients, MHC I expression can be mediated by administering STING agonists, retinoids, and TNF-α, which activate the NFκB pathway as well as MEK and STAT3 inhibitors (currently investigated in clinical trials) [122].

### 7.5. Immune Checkpoint Inhibitors

Cytotoxic T-Lymphocytes Antigen 4 (CTLA4) regulates the activation of T and NK cells. Upon binding with CD80 and CD86 on Antigen-Presenting Cells (APC), CTLA4 inhibits the response to antigen presentation in both T cells and NK cells. Anti-CTLA4 therapy has shown promising results in both animal models and patients by reversing immune evasion in several tumor types, including unresectable metastatic melanoma [123]. Increased PD-L1 expression by metastatic and dormant cells, as well as the immune-modulatory effects exerted by T_regs_ and MDSCs through the secretion of TGF-β1 and IL-10, play a role in compromising the anti-tumor activity of NK and adaptive T cells. PD-L1 blockade restores NK-cell mediated killing of dormant cells before they can be reactivated and drive metastatic recurrence [124]. From a broader angle, consistent monitoring of NK- and T-cell abundance and response in cancer patients with MRD could help predict disease progression. Potentiating both NK- and T-cell-mediated responses against metastatic and dormant tumor cells may convert advanced metastatic disease into a more manageable chronic condition [78].

### 7.6. Clinical Trials

Several initiatives have been trying to implement clinical trials for LB in oncology [125]. In 2015, Cynvenio Biosystems obtained approval for a clinical trial of their LB system (LiquidBiopsy) for the detection of CTCs and NK cells in women affected with metastatic triple-negative breast cancer [126]. Recently, a clinical trial has been approved to test the applicability of the combination of multi-parametric Magnetic Resonance Imaging (MRI) with LB in detecting CTCs in breast cancer patients (UMC—Utrecht Medical Center, Netherlands) [127]. In France, a new clinical trial aimed at determining the accuracy of LB in predicting response to first-line immunotherapy in patients with NSCLC is currently recruiting participants [128].

Numerous clinical trials involving immunotherapy of metastatic cancer are currently ongoing; however, only a few have been specifically designed to study the involvement of the innate immune system in metastatic cancer [129]. Among the trials analyzing the role of the innate immune system in therapeutic response and disease progression, only one appears to be focused on patients affected by metastatic breast cancer (University Hospitals of Valencia and Murcia, Valencia and Murcia, Spain). Looking at a broader perspective, several current trials are designed to determine the role of specific immune subtypes in response to therapy, including PD-L1 expression on T_regs_ in prostate cancer (Yonsei University, Seoul, Korea), the role of NK cells in solid tumors (efficacy of AFM24 in advanced cancers—UCLA, Los Angeles; Dana Farber, Boston; Vall d’Hebron Institute of Oncology, Barcelona; Institute of Cancer Research—Royal Marsden, London, UK) and colorectal cancer (Hôpital Maisonneuve-Rosemont, Montreal, QC, Canada), and the characterization of the immune profile of breast (Chu Grenoble, Grenoble, France) and head and neck cancers (IRCCS Neuromed, Rome, Italy).

Two clinical trials involving the use of NK cells as a curative therapy for metastatic cancer are planned to start (National Institutes of Health, Bethesda; and Hopitaux de Paris, France), with the study coordinated by the NIH focusing on targeting metastatic head and neck or gastric cancer with PD-L1 CAR-NK cells [130] and the trial in Paris involving NK cells as neoadjuvant therapy in metastatic melanoma patients [131]. Thus far, more than 150 clinical trials focused on correlating NK-cells activity and survival in patients with advanced disease (prostate and colorectal cancer) or exploiting their natural ability to target and attack tumor cells have been completed. Infusions of NK cells alone (e.g., ovarian cancer) or in combination with different treatments (Everolimus, Trastuzumab, and Cetuximab for breast cancer, NSCLC, gastrointestinal cancer) have been assessed in several trials. Combinations of immune-checkpoint inhibitors with autologous NK-cell infusions have also been evaluated (with anti-PD-1 or IL-12 in advanced renal cell carcinoma and melanoma) [132]. Most of the aforementioned trials have not disclosed their results yet and have been conducted on a small scale. Combination of NK cells, IL-12, and standard chemotherapy showed dismal results in melanoma and kidney cancer. Patients with ovarian, peritoneal, and breast cancer, however, seemed to benefit from the combination of IL-2 (6 doses), NK cells, 1 mg/Kg Methylprednisolone, and CsA (cyclophosphamide, fludarabine, and cyclosporine A), with 43% of subjects surviving at least 1 year after receiving the first treatment. In a different trial, metastatic breast cancer patients showed a promising outcome, with the majority of subjects (83%) surviving over 100 days after receiving an infusion of NK cells with or without total body irradiation. Treatments involving NK cells and Cetuximab or Everolimus, on the other hand, have not shown encouraging results.

Several clinical trials have recently been completed focusing on the reactivation of an anti-tumorigenic response in tumor-infiltrating macrophages. Vaccines with engineered tumor cells actively secreting pro-inflammatory cytokines have been tested against a panel of metastatic tumors, including breast (autologous cancer cells expressing GM-CSF, Dana-Farber Cancer Institute and Brigham and Women’s Hospital in Boston; GM-CSF in combination with chemotherapy, NCI, Bethesda) and colorectal cancer (GVAX in combination with Cyclophosphamide, Johns Hopkins, Baltimore—results pending). Injection of autologous cancer cells expressing GM-CSF appears to cause late cancer progression (13 months) in 8.3% of participants and absence of progression in another 8.3%, while the remaining subjects experienced cancer progression within 4 months [133]. A vaccine activating melanoma-associated macrophages via AV-MEL-1, in combination with anti-PD-1, is currently recruiting patients (Hoag Hospital, Irvine, CA, USA) [134] and so is a new study aimed at developing imaging systems to detect M2 TAMs in metastases from melanoma (Centre Hospitalier Universitaire Vaudois, Lausanne, Switzerland) [135].

A clinical trial studying the effects of a neutrophil re-activating vaccine in metastatic breast cancer is currently recruiting participants. This vaccine is based upon a modified measles virus carrying the sequence of the neutrophil-activating protein from *Helicobacter pylori*-MV-s-NAP (Mayo Clinic in Rochester, MN, USA) [136]. Table 1 summarizes the recent advances in the development of therapeutic and diagnostic tools involving the innate immune system in cancer.

## 8. Clinical Implications and Conclusions

Metastatic dissemination is an inevitable consequence of many cancers and, due to its complexity, it has become increasingly important that we determine what promotes DTC dormancy and the mechanisms that result in the eventual escape from dormancy. Therapies targeting primary tumors, while often efficacious, frequently fail to eliminate DTCs, resulting in eventual recurrence and patient mortality. The unpredictable nature of metastatic recurrence remains a clinical challenge, with a substantial impact on patient quality of life [139]. Besides the design of novel therapeutic regimens, detecting micro-metastatic clusters of dormant cells before they reactivate is of paramount importance. Ideally, patients could be screened for the presence of dormant cells, and once identified, the dormant cells could be targeted using several approaches that would reinforce strong anti-tumor responses by both innate and adaptive immune cell subtypes. To this end, strengthening the immune system response in patients with increased risk of recurrence might represent a more precise tool for targeting DTCs while preserving non-cancerous cells. However, activating the immune system in patients recovering from debilitating diseases like cancer may be challenging. Side effects may range from milder symptoms, such as headaches and hypertension, to the establishment of life-threatening conditions like kidney necrosis and autoimmune toxicity that eventually compromises the functionality of otherwise healthy tissues [140].

While research continues to provide insight into how the detection of tumor-permissive cytokines and immune cell subtypes within the blood of patients in remission can warn of an impending recurrence, additional studies of how the immune system contributes to the escape of a tumor cell from dormancy are clearly needed. Effective diagnostic approaches could rely on detecting clusters of dormant cells by using markers bound to either endostatins or monoclonal antibodies with specific affinity to dormant cells. Detecting clusters of tumor cells and tumor-supporting immune cells within the bloodstream of patients could inform about the dissemination stage of the primary cancer and provide insights about MRD and the possible reactivation of dormant cells in response to pro-inflammatory stimuli. At the same time, the frequency and subtype of immune cells detected inform clinicians about patient prognosis, with the presence of NK cells and non-exhausted CD8^+^ T cells suggesting a more favorable outcome and potential response to immunotherapeutic regimens.

Our current understanding of DTCs dormancy and interactions with the immune system provides us with two potential approaches: tracking down and eradicating all dormant cells by triggering the patient’s immune system or by inducing an irreversible dormant state throughout the patient’s life. In eligible patients, targeting dormant cells by combining molecules that trigger anti-tumor immunity, like IFN-β agonists or repressors of TGF-β1 signaling, with immune checkpoint inhibition might represent a successful strategy to control and eventually eradicate dormant cell niches. Re-programming the immune system to fight dormant tumor cells could grant access to compartments usually precluded to traditional treatments and help develop a more personalized therapeutic approach that takes into account specific features of the patient’s immune system, including the anti-tumorigenic potential of circulating innate and adaptive immune cells. Such a plan will require more research to identify unique and shared features of the systemic and localized immune system of patients with different types of cancer. As different subtypes of cancer tend to seed specific organs, molecular analysis of dormant cell niches depending on the type of cancer and the secondary organs will be necessary. Going back to the “Seed and Soil Hypothesis” previously described, a better understanding of the factors leading a cell from a specific primary tumor towards a secondary tissue and influencing its behavior is further required.

## Figures and Tables

**Table 1:** Summary of the clinical trials and pre-clinical studies focusing on the innate immune system for therapeutic and diagnostic purposes currently under investigation in the context of metastatic tumors.

Approach	Purpose	Focus Area	Phase	Refs
PD-L1 CAR-NK cells	THERAPY	Therapeutic targeting of CTC and dormant cells	phase I	[130]
NK-based neoadjuvant therapy	THERAPY	Therapeutic targeting of CTC	phase I	[131]
Vaccines increasing pro-inflammatory response in macrophages	THERAPY	Prevention/targeting of CTCs	phase I and II	[133]
Detection of M2 TAMs in metastases from melanoma	DIAGNOSIS	Detection of micrometastases and anti-inflammatory priming of the tumor immune microenvironment	phase I	[135]
Vaccine activating pro-inflammatory response in neutrophils	THERAPY	Stimulation of neutrophil-mediated pro-inflammatory response against CTC	phase I	[136]
Immune profiling of metastatic cancers (innate immunity)	DIAGNOSIS	Innate immune profiling of metastatic tumors	phase I	[129]
CAR-T cells targeting NKG2D ligands on tumor cells to prevent NKs exhaustion	THERAPY	NK-cells-mediated dormant cells clearance	phase I	[137]
Integrin α5-targeting mAb: Intetumumab	THERAPY	Prevention of dormant cells reactivation	phase I and II	[97]
Integrin α5-targeting mAb: Abituzumab	THERAPY	Prevention of dormant cells reactivation	phase I and II	[98]
Integrin α5-targeting mAb: Volociximab	THERAPY	Prevention of dormant cells reactivation	phase I and II preclinical models	[99,101]
Endostatin: endogenous antagonist of integrin α5	THERAPY	Prevention of dormant cells reactivation	phase II, III, IV	[102]
STING agonists: reactivation of IFNβ pathway (prevention of EMT, activation of pro-inflammatory response, prevention of exit from dormancy)	THERAPY	Activation of pro-inflammatory response against CTC and dormant cells	phase II preclinical models	[104,111,112]
TEW-7197: inhibitor of TGFβ-1 pathway	THERAPY	Activation of pro-inflammatory response against CTC and dormant cells	phase I and II preclinical models	[115,116]
Immune checkpoint inhibitors (anti-CTLA4, anti-PD-L1)	THERAPY	Suppression of immune evasion mechanisms mediated by immune checkpoint expression on CTCs and dormant cells	phase III preclinical models	[123,124]
RGD peptides targeting integrin α5 (β1 and β3) overexpressed on tumor cells	DIAGNOSIS	Detection and targeting of micro-metastases	phase I preclinical models	[103,138]
Multi-parametric MRI with LB to detect CTC in the blood	DIAGNOSIS	Detection of CTC in the blood	phase I	[127]
LB for the detection of cf-DNA predicting response to immunotherapy	DIAGNOSIS	Assessment of response to immunotherapy	phase I	[128]
